# Experiences of trans women who have undergone gender affirmation surgery: a constructivist grounded theory

**DOI:** 10.3389/fsoc.2025.1544906

**Published:** 2025-05-02

**Authors:** S. T. S. Dharsheni, B. Sivakami

**Affiliations:** Division of English, School of Social Sciences and Languages, Vellore Institute of Technology, Chennai, India

**Keywords:** gender affirmation surgery, experience, affirmative care, advocacy, resilience

## Abstract

**Introduction:**

Gender Affirmation Surgery often referred to as ‘Nirvana’ in the transgender community in India is a major process that most trans women undergo. It is practiced in the traditional way as, the thayamma operation (midwife procedure) where the elderly transwoman cut off the penis and testicles without any anesthesia or medical supervision. However, the medical procedure of GAS in India is a recent advancement, which is accessible only to a few, the procedure that most trans women have undergone is penectomy (removal of the penis) and orchiectomy (removal of the testicles).

**Aim:**

This study aims to explore the experiences of trans women who have undergone gender-affirmation surgery (GAS) during the period 2005–2023 in Chennai, India.

**Method:**

In academia, research papers discuss the medical aspects of gender affirmation surgery but the experiences of the individuals undergoing this surgery remain under-researched and under-documented, which was the main reason to apply a constructivist grounded theory approach. Seventeen trans women from various non-governmental organizations (NGOs) in Chennai, India participated in the in-depth interviews, which were conducted in two sessions, to gain insights into their lived experience of undergoing gender affirmation surgery.

**Result:**

This study, using initial coding, focused coding, and theoretical coding, reveals both the positive and negative aspects of the surgery experiences of trans women using the five focused codes: decision to undergo surgery, support systems, healthcare experiences, postoperative outcomes, and impact on identity and well-being.

**Discussion:**

The study highlights the resilient nature of trans women in India, who despite facing adversity, become advocates for better healthcare and social understanding. The findings emphasize the importance of inclusive policies and practices that address the unique needs of trans women.

## Introduction

Trans women in India often face significant challenges when seeking gender affirmation procedures. Challenges arise from societal attitudes and lack of access to proper healthcare services. This leaves trans women vulnerable to unsafe medical practices, increasing the risk of physical complications and exacerbating the psychological distress that accompanies their gender transition.

Gender Affirmation Surgery includes procedures such as vaginoplasty, clitoroplasty, penectomy, orchidectomy, and metoidioplasty (Agochukwu et al., 2022; Chen et al., 2019; Selvaggi and Bellringer, 2011). Prior to the surgery, individuals usually undergo psychotherapy and hormonal therapy as well (Selvaggi and Bellringer, 2011). These surgeries are crucial in helping trans women achieve gender congruence (Breslow et al., 2021). In this paper the term GAS, is used to cover gender affirming practices of transwoman in Chennai ranging from traditional castration to surgeries taking place in unsafe clinics and the recent Gender affirming surgeries in hospital. By bringing all the affirming practices under the term GAS, this study collectively explores transwoman’s experiences with GAS.

In Indian hijra culture, “Nirvana” involves the removal of male genital organs and the transformation into a woman’s body through castration, along with a series of rituals (Tripathi, 2015). It refers to the ritualistic castration or shedding of male genitalia, a process that signifies both physical transformation and spiritual liberation, often used as an umbrella term within Indian transwomen to refer to all forms of surgical transition.

This practice significantly affirms hijra identity due to its physical visibility and ceremonial symbolism (Brinda and Gayathri, 2023). Historically, trans women in India have resorted to traditional methods for gender affirmation. Thayamma or “Dai Amma” operation (Srivastava et al., 2023), is a crude midwife procedure involving the removal of the penis and testicles without anesthesia, which reflects the extreme measures trans women have taken to achieve bodily autonomy (Petry, 2015). The journey of trans women to achieve alignment between their gender identity and physical bodies is fraught with various social and medical challenges.

More commonly, the majority of trans women undergo surgeries in unauthorized small clinics due to financial constraints and other factors. They pay nominal fees for procedures like penectomy (removal of the penis) and orchiectomy (removal of the testicles) for the removal of the penis and testicles without constructing a neo-vagina. The genital nullification surgery which is the combined procedure of penectomy and orchiectomy is also a part of the gender-affirmation surgery. Unfortunately, these clinics lack proper psychological support and adequate medical facilities. Autobiographies of prominent transwomen from India document the reality of this procedure. Living Smile Vidya in her (2013) autobiography describes the hospital where she underwent the surgery as a “slaughterhouse” Vidya (2013). Revathi on the other hand writes, “I had to put up with all these painful procedures if I wanted to become a woman” (Revathi, 2010:63). With increased medicalization and legal recognition of transgender rights, many trans women now seek Gender Affirmation Surgery (GAS) in private hospitals, where procedures are conducted under better medical supervision.

Scholarly literature has largely overlooked the experiences of transwomen who have undergone gender affirmation surgery. Studies in this area are predominantly on the surgical aspects, mental health, HIV/AIDS, stigma, and discrimination. Research on the experiences of transwomen who have undergone GAS in the Indian context is relatively new. Documenting these experiences will aid in identifying the challenges trans women encounter when accessing healthcare and quality of service, thereby informing targeted interventions and policies to improve the overall healthcare experience for transwomen undergoing GAS.

Numerous medical research articles discuss different surgical aspects of Gender Affirmation Surgery. Petry (2015) discusses various transformative processes for constructing the female body, including behavior adaptation, posture modification, voice modulation, hormone use, vaginal canal dilation, and surgical complications. Pariser and Kim (2019) examines the techniques involved and the outcomes of vaginoplasty. Saltman et al. (2023) compares the outcomes of Transgender orchiectomy (TGO) to cisgender orchiectomy (CGO) for nononcologic indications. Chen et al. (2019) studies the overview of surgical techniques used in gender-affirmation genital surgery. However, these procedures remain accessible only to a few, reflecting ongoing healthcare inequities (Subbaraj and Tirougnanassambandamourty, 2020). Gupta (2022) notes a significant rise in the prevalence of gender incongruence in India, with multiple centers now performing a high volume of gender-affirmation surgeries, indicating an increased demand for these services.

Stigma and discrimination in healthcare settings have significant impacts on transgender individuals. Poteat et al. (2013) highlight that such negative experiences reinforce the power and authority of medical providers, even when they harbor uncertainty and ambivalence about transgender patients. Similarly, a comprehensive review of transgender health services in India reveals that the transgender community faces significant discrimination and a high burden of disease, including mental health issues and HIV/AIDS (National AIDS Control Organisation and Ministry of Health and Family Welfare, 2021). Transgender individuals often struggle with the psychological challenges that are inherent to their journey toward self-discovery and acceptance. The mental health requirements for gender affirmation surgery can be complex and varied (Marra et al., 2024). Calhoun et al. (2022) emphasizes the importance of social support, finding that acceptance from support groups involves a process of change involving the self, relationships, and society, upheld through the willingness of the support person. Subbaraj and Tirougnanassambandamourty (2020) indicates that gender reassignment surgery significantly improves the quality of life, particularly in the psychological health domain.

Sullivan (2020) conducted a constructivist grounded theory study to understand nurses’ gender transition experiences. The resulting theory, ‘Becoming Myself,’ explains the social processes nurses utilize during the transition and the barriers and facilitative factors they encounter in both personal and professional contexts. Extending this line of inquiry, Mathai and Pradeep (2022) study sheds light on the socio-cultural, economic, and health aspects of Hijras’ lives, challenging dominant cultural narratives surrounding this ethnic group. Raghuram et al. (2024) explored the experiences of 63 transgender individuals in India accessing routine healthcare services, revealing intersectional challenges and negative impacts. Applegarth and Nuttall (2016) highlight the complexity of the therapeutic experience for transgender clients, emphasizing the need to understand their lived experiences in therapy. Nadan (2022) research focuses on the challenges faced by secular middle-class Jewish parents of transgender emerging adults in Israel, shedding light on their concerns for their children’s well-being and the support they need to provide. Persson Tholin and Broström (2018) study in Sweden reveals the significant barriers transgender and gender-diverse individuals encounter when accessing non-transition-related healthcare. Similarly, González and Veale (2024) research identifies the lack of trans-competent providers in New Zealand and experiences of mistreatment contributing to health disparities.

Mitchell and Howarth (2009) call for a comprehensive study on the economic position, experiences, and needs of the transgender population, emphasizing the importance of both quantitative and qualitative research to capture the diverse experiences of trans people. Understanding the experiences of individuals undergoing gender affirmation procedures is crucial, as it reveals the intricacies of gender transition and becomes increasingly relevant as more people seek to align their physical appearance with their gender identity. Through a constructivist grounded theory approach, this research draws a model to display the complex interplay of societal pressures, gender norms, and experiences of trans women.

## Research question

What are the experiences of trans women who have undergone gender-affirming procedures, including traditional castration by Thayamma within the trans women community, informal surgical procedures in unauthorized clinics, and hospital-based gender-affirming surgery with medical supervision?

## Materials and methods

This study employs a qualitative approach, Constructivist Grounded Theory by Kathy Charmaz (2006) to explore the experiences of trans women from Chennai who have undergone gender affirmation surgery. CGT highlights the co-construction of meanings between researchers and participants, making it particularly suitable for exploring the life experiences of marginalized groups. Instead of starting with a predefined theoretical framework, CGT allows theories to emerge out of data, ensuring a conceptual understanding of the stories of trans women undergoing gender-affirming surgery (GAS). However, the themes that emerged in this study resonate with broader trans-feminist perspectives, offering a critical lens to interpret the findings.

The study collected data from three non-governmental organizations that support trans women in Chennai. Seventeen participants were identified using snowball sampling. The researcher first interacted with NGO senior leaders, gaining their trust by explaining the academic intent and motif of the study. Projective techniques such as thematic apperception using images of familiar trans activists and news clippings related to transwomen are employed. The discussion of the autobiographies of trans women was also used as prompts, facilitating transparent communication. The researcher acknowledged her positionality as a cisgender academic working in transgender studies with prior experience conducting a master’s dissertation on trans women in collaboration with one of the participating NGOs.Although this familiarity aided in building trust, it had no influence on participant selection because the researcher had no prior relationships with other NGOs or participants. The study focused on participant-led discussions, focusing on their lived experiences as allies to the trans community. Participants were chosen only based on their self-identification as trans women, not other genders or diverse sexual identities. To guarantee comfort and safety, interviews were held at venues selected by the participants, mostly inside NGO buildings.

Data was gathered in two phases between April and May 2024, followed by additional theoretical sampling in July 2024. An interview guide comprising semi-structured interviews was developed and verified in consultation with subject experts and community members, ensuring relevance and sensitivity to trans experiences. An official signed consent of my presence at the NGO was received and individual consent from each participant was obtained emphasizing autonomy over what they shared. Participants were informed of their rights to opt out of the interview if they felt uncomfortable at any moment. The questions were translated into the regional language Tamil. The interview duration per person was approximately 40 to 45 min. The interview transcripts were translated into English, internally validated, and further validated with the corresponding non-governmental organization. This process ensured the data’s accuracy and reliability. Later, memos were written after each interview as part of the analysis to record initial thoughts and emerging themes for each participant, providing a detailed understanding of individual experiences, and a new set of questions was prepared for the second round of interviews.

Memos were also written to identify overarching themes and patterns. All questions and areas of inquiry were documented in the memos, allowing for a comprehensive and transparent record of the research process. Furthermore, insights gathered from external sources, such as relevant literature and expert opinions, were integrated into the memos. To enhance clarity conversational style, clear headings, and concise pointers were utilized to organize the memos.

Although there was no systematic post-interview follow-up process after the interview, the NGOs continued to offer community support to participants such as peer counseling, emotional support, awareness and better access to healthcare. For practical reasons and issues of participant privacy, direct follow-ups were not done. No further resources like psychological, legal, or medical were offered directly by the researcher. The participants did not expect any financial compensation, it was discussed before the interviews. Yet, in exchange as a non-monetary return, the researchers will share the study’s results and final paper with the NGOs and participants.

### Initial coding

Initial coding was conducted by carefully reading and analyzing each transcript, codes were assigned to individual words, phrases and sentences that captured the essence of the participants’ experiences. During this process memos were used to record thoughts, questions and insights. A line-by-line coding approach was used, to identify early analytic ideas and potential theoretical directions (Charmaz, 2006). This helped to track the analytical journey and identify emerging patterns. As emphasized by Charmaz, initial coding was approached without preconceptions (Tables 1 and 2).

**Table 1:** Initial codes and subcategories of Round: 1 interview.

Initial Code	Sub-Category
Surgery Decision	Sharing of Experience
	Promotional Questions (Influence) Social Pressure (inside and outside)
	Coercion
	Personal motivation
Emotional Support	No Family Support
	Guru Support
	Self-Support
	Community Support
	Emotional Support of Medicinal Practitioners
Financial Support	Self Funding
	Partial Funding
	Insufficient Finances
	Support from guru (community)
Surgery Setting	Illegal Surgery location
	Inadequate facilities and infrastructure
	Adequate facilities
	Inadequate pain management (overdose/underdose)
Post Surgery Complication	Unprofessional behavior by medical staff
	Inhumane treatment post-surgery
	Suspect unqualified medical personnel
	No post-operative care
	Lack of follow-up from hospital
	Side effect
	Re-surgery
Caregiver Support	No caregiver
	Guru as caretaker
	Negative caregiver experience
	Negligence
	Poor choice of hospital
	Family
	Self
	Community
Pre-Surgical Information	No prior Information
	HIV test only
	Limited test
	Inclusion of all test
	Potential risk/complication was not highlighted
	Lack of knowledge of informed consent before signing
	Availability of food prescription
	Non-availability of food prescription
Mental State	Fear
	No Fear
	Live or Die Attitude
	Anxiety/Panic
	Excitement /Anticipation
	Pray
	Self-determination
	Lonely
	Mental block

**Table 2:** Initial codes and subcategories of Round: 2 interview.

Initial Code	Sub-Category
Physical and Mental Changes (After Surgery)	Satisfaction
	Feeling better
	Look and feel like a woman (Appearance)
	Naturally appears like woman
	Change in body structure
	Gaining weight
	Lack of stamina
	No physical pleasure
	Mentally woman
	No facial hair/body hair
	Body language
Challenges	Discrimination by health care providers
	Pressure to confirm to mainstream society
	Hegemonic Gender Roles at relationship, workplace, home
	Social Isolation
	Social pressure
	Personal Struggles
	Identity conflict
Personal Relationship	Relationship Strain
	Alienation
	Support
Social Reaction	Positive
	Negative
Coping	Community Support
	Personal Resilience
Decision	Community Influence
	Desire to be beautiful
	Emotional readiness
Support Network	Community Support (Guru)
	Peer Support (Trans Community)
Medical Treatment	Discrimination
	Lack of professionalism/respect
	Professionalism
Reflection and learning	Regret
	Recent advancements (Awareness) (sharing it with future generation)
	Advocacy
Post-operative care	No post-operative care instructions
	No follow-up appointments
	Follow up
	Lack of psychological support
	Complications
	Re-surgery
Surgery Preparation	No Physical Preparation
	Mental Preparation
	Financial planning
Recovery Period	Physical Recovery
	No mentions of emotional recovery
Additional Medical Procedures	Hormone Therapy
	Cosmetic procedure-Silicone breast implant
	Laser
Mental Health Needs	Pre-surgery anxiety
	No mention of therapy pre- and post-surgery
	Depression
	Need for therapy but no finances
	No access to therapy
	To learn coping mechanism
Dysphoric After	Emotional Stability
	Residual dysphoria
	Insecurity

### Focused coding

Following the initial coding of the round 1 interview, focused coding was conducted by manually reviewing and combining the significant codes. The most frequent initial codes were selected and analyzed to identify patterns, relationships, and themes. A table was created to systematically organize the data. Through this iterative process, the most prominent categories were identified, developed, refined and integrated into a cohesive theoretical framework (Charmaz, 2006).

After the initial interviews, a second round interview were conducted using projective techniques such as thematic apperception and ink blots to reveal the underlying emotions of the participants. The questions for this round were developed from the responses of the first round of interviews and memos. Following the second round of interviews, the analysis was refined through a process of reexamining the codes, adding new codes based on the new data and updating memos. This led to theoretical saturation, where no new themes, concepts, or codes emerged from the data.

### Theoretical coding

Theoretical coding helps incorporate core categories into a cohesive model. This stage focused on integrating categories to provide a holistic understanding of trans women’s experiences with gender affirmation surgery.

### Validating the conceptual model

Using the same interview guide, two more participants were interviewed, and their responses were consistent with the findings. This step helped ensure that the model was consistent.

## Results

The research reveals the following as focused codes: decision to undergo surgery, support systems, healthcare experiences, postoperative outcomes, and impact on identity and well-being. The results highlight the positive and negative aspects of their experiences, offering insights into the complexities and challenges they faced. The decision to undergo traditional castration, an operation with limited medical facilities, or proper gender reassignment surgery is influenced by various factors, as revealed through interviews. These factors include socioeconomic status and the influence of seniors. Specifically, individuals who opt for underprivileged treatment, such as non-medical castration or castration at unapproved clinics, often do so due to lack of support, awareness, and education. In contrast, transgender individuals who have undergone modern gender affirmation surgery tend to have better education, community support, and awareness.

### Decision making

The decision to undergo Gender Affirmation Surgery is influenced by various factors, including transition motivation, social influence, emotional readiness, and the desire to be beautiful. Participants shared their experiences, highlighting the intricacies of this decision-making process.

Participant:1 described her experience of gender incongruence, leading to her decision to undergo surgery:

*I felt mentally different, and I did not want the physical thing, when there was this realization in me I opted for the surgery* (Participant 1).

This experience of gender incongruence is a common theme among individuals considering GAS. However, the decision-making process is not solely individual; it can be influenced by various external factors, including social norms and peer pressure. Dean Spade (2006) states that trans bodies are subjected to a norm-producing medical discipline, where surgery is positioned as the only way to be recognized as a “real” woman. This perspective resonates with Participant 2’s experience, where she was coerced into undergoing surgery before she was emotionally or medically ready:

*I was threatened to undergo the surgery. I didn't want to go through the surgery because I was aged and had high blood pressure and diabetes even before going to the surgery, but I was forced into it* (Participant 2).

Spade’s critique that the reification of gender norms within trans healthcare creates a conditional form of acceptance where gender is validated through physical modification rather than self-identification applies to peer influence within trans communities, where gender affirmation surgery is often framed as a necessary milestone to attain complete gender recognition. Participants 3 and 4 described how seeing others undergo surgery created an implicit pressure to follow the same path:

…few who underwent the sex reassignment surgery discussed about it … and others got influenced … We all had the desire to transform our bodies too. However, when someone gets the surgery and others don't, it creates a question like, 'Haven't you done it yet?' This question induces ego and makes people go for it, not by force but by desire after seeing the seniors of the community who got operated (Participant 3).

People within my community including my transwomen friends have asked me, you look good like a woman, but why haven’t you done it (Surgery) yet? (Participant 4).

For some individuals, the desire to be beautiful also plays a significant role in the decision-making process. Participant 5’s decision was primarily driven by this desire to attain an ideal feminine body,

*“I wanted to become a beautiful woman with a feminine body, free of facial and body hair, and with breasts”* (Participant 5).

This reflects the pressures of hegemonic femininity (Connell and Messerschmidt, 2005), where trans women must conform to cisnormative beauty standards to be socially validated. These pressures make gender affirmation surgery less of an individual choice and more of a coerced necessity for social survival (Spade, 2006).

Participant 6 expressed satisfaction with the surgery outcome,

*I am completely satisfied with the outcome of the surgery. I now look and feel like a woman in my daily life. The surgery has brought me satisfaction, and I am happy to have transitioned to a woman* (Participant 6).

Participants reported a better understanding of their gender identity as a positive outcome, which aligns with existing literature emphasizing the importance of gender congruence for mental health (Thoma et al., 2023). However, uncertainties about the decision and anxiety over potential outcomes were common negative experiences, highlighting the need for comprehensive pre-surgical counseling, support and necessary postoperative care (Coleman et al., 2022). Participant 4 shared anxious feelings after deciding to undergo surgery, highlighting the importance of support and guidance throughout the process.

*After I took the decision to undergo the surgery, I was little anxious thinking of the surgery… will it be successful or not? it was not fear but a kind of anxious feeling of the major transformation ahead* (Participant 4).

Similarly, Participant 7 underwent surgery of her own free will, but experienced doubts regarding her decision, which is often due to lack of support.

*I underwent the surgery of my own free will, without pressure from anyone. However, there was a mild oscillation accompanied by nervousness as I was alone opting for the surgery without any support from family or fellow trans women* (Participant 7).

This portrays the way that decision-making regarding GAS is shaped by larger social structures since trans women have to resist medical expectations, community pressures, and internalized gender norms when deciding about their bodies.

### Support system

Support systems including family, friends, partners, community support, and self-support, play a crucial role in the experiences of trans women undergoing GAS. However, Namaste (2000) and Valencia and Zhuravleva (2019) states, trans individuals are often structurally erased from mainstream family and social networks, forcing them to rely on chosen families and trans communities for survival. The majority of the participants had the trans community as their support system. Support from family and cis friends was relatively low. However, few transwomen had the support of their partners. Positive experiences were marked by community engagement and connection, which provided emotional and practical support during the surgical process. This indicates a need for broader social acceptance and targeted interventions to foster supportive environments for trans women. Rejection from family and discriminatory experiences were significant negative aspects, underscoring the pervasive social stigma and negative mental health outcomes, such as depression faced by trans individuals in India. Chakrapani et al. (2018) and Namaste (2000) notes that this exclusion is not just personal but structural, reflecting how cisnormativity conditions family acceptance in adherence to gender norms, leaving transwomen to economic and emotional vulnerability.

Participant 8’s experience highlights the challenges of lacking family support.

*Initially, when I was about to go for the surgery, my family members came to know about it, and it caused a lot of problems. Tackling all that, I went for the surgery. There was no support from my family’s end. I borrowed money here and there and felt left alone* (Participant 8).

### Healthcare experience

The healthcare experience of trans individuals undergoing Gender Affirmation Surgery encompasses the following categories: access to services, provider interaction, facility and infrastructure, nature of the practitioner, financial considerations, and location and setting of the surgery. The study revealed both positive and negative experiences. Positive experiences included reaffirmation of their gender identity and supportive interactions with healthcare providers. However, several concerns emerged, including financial constraints, improper procedures, involvement of unqualified practitioners, and anesthesia issues. This aligns with previous research indicating systemic barriers and disparities in healthcare access for trans individuals (Safer et al., 2016).

Participant 4’s experience highlights the lack of proper facilities in hospitals, specifically in the transgender ward,

*The surgery place was a little unhygienic… that didn't matter because I anyway wanted to get done with the surgery* (Participant 4).

In contrast, Participant 6’s experience reveals the devastating consequences of improper procedures and unprofessional behavior exemplifying iatrogenic violence (Atuk, 2024) where healthcare settings become sites of dehumanization.

*My surgery was not performed properly and my I was in a life-or-death situation. The unprofessional behavior of the doctors were evident throughout my surgery period as they were chit-chatting among themselves about trivial things… Even though I was under anesthesia, I could hear them giggling and talking about food and other daily routines* (Participant 6).

Similar to this is Participant 9’s experience, it highlights the risks of unqualified practitioners,

*The woman who performed my surgery is not a qualified MBBS doctor, but an assistant who has observed doctors performing surgeries and now performs them herself… Many people had to undergo a second surgery due to the inadequacy of the medical facility* (Participant 9).

Erickson-Schroth (2014) highlights that healthcare disparities for trans individuals are influenced by intersecting factors, including economic status and access to medical knowledge. Participant 7’s case reflects how gendered neglect operates in trans healthcare, where even basic pre-surgical screenings are overlooked,

*I wasn't given any information about the procedures before undergoing the surgery… Doctors had performed the surgery properly, but what became the problem was, that I had diabetes before the surgery which I was not aware of, and they didn't check my blood sugar levels… hich led to complications* (Participant 7).

The experiences of transwomen in healthcare illustrates how necropolitical power does not just operate explicitly through violence but also as systematic neglect and medical exclusion. Mbembe and Corcoran (2019) pushing vulnerable populations to life-threatening risks.

### Postoperative care

Post-operative care is critical for the recovery and long-term well-being of trans women. Positive experiences were associated with supportive care from the community, facilitating physical and emotional recovery. However, many participants reported insufficient postoperative care, lack of follow-up with doctors, long-term complications, and limited access to therapy. These negative outcomes highlight gaps in the continuity of care and the need for integrated comprehensive care that provides ongoing support post-surgery (Carlson et al., 2021).

Participant 3’s experience underscores the importance of informed consent and adequate post-operative care.

*I underwent surgery without being informed about the procedures involved… I was not aware of the post-effects of the spinal anesthesia. As a result, I am now unable to sit straight for more than 30 minutes, have a hunchback, and severe pain in my spine. I am currently undergoing physiotherapy for both leg and spine issues* (Participant 3).

Trans individuals have the sole right to make decisions regarding their own bodies and that no political, medical, or religious authority should violate their bodily integrity (Koyama, 2003). Yet, participant experiences reveal a striking contradiction between this principle and the reality of medical neglect.

Participant 9’s experience highlights the need for adequate post-operative care. She was discharged from the hospital without proper post-care measures and faced mockery from strangers, adding to her distress.

*After the surgery, the doctors discharged us even before the removal of the urine bag. They didn't provide any post-care measures…I was also subjected to mockery by strangers on my way back from the surgery* (Participant 9).

Participant 3 also emphasizes the importance of self-care and support during the recovery period.

*For the first 15 days, I had a caretaker attending to me, but after that, I had to do things on my own with the intention of learning to take care of myself and being able to care for others who undergo surgery in future* (Participant 3).

Participant 3 had concerns regarding the competence of her surgeon.

*I felt pain even after being given anesthesia. As a result of a mistake during the operation, I now have a problem with my right leg* (Participant 3).

### Identity and wellbeing

The impact of GAS on identity and well-being is multifaceted. Positive outcomes included increased satisfaction with the body, alignment with gender identity and enhanced self-esteem. These benefits are consistent with research showing improved mental health and quality of life following gender-affirming procedures (Hughto et al., 2020). Brinda and Gayathri (2023) conclude, that despite medical and specific surgical complications, satisfaction with transition is high. Nevertheless, some participants experience body dissatisfaction and insecurity due to fear of rejection. This suggests that while GAS can significantly improve well-being, comprehensive support addressing psychosocial aspects is essential for long-term positive outcomes.

Participant 5 reports satisfaction with her surgery outcome despite experiencing post-surgical complications.

*I am experiencing back pain, which is expected for trans women post-surgery due to the injections. However, I am very satisfied after undergoing the surgery* (Participant 5).

Trans feminist thinkers assert that individuals can define their identities and expect respect from society (Stone, 1992). It challenges all women, including trans women, to interrogate internalized gender norms, simultaneously acknowledging that liberation does not necessitate the rejection of all patriarchal markers of femininity (Koyama, 2003).

### Reflection and advocacy

Reflecting on the experiences, the resilience and advocacy efforts of trans women amid adversity are revealed. The journey through GAS is not merely a medical process but a personal and social transition requiring strong support systems and affirmative healthcare practices. Advocacy for trans rights, improved healthcare access, and social acceptance are critical in addressing the challenges highlighted in the results.

Participant 2 emphasizes the importance of self-identification and respect for trans women’s identities stating,

*I decided that no other transwomen should go through similar pain … they should be given counseling and told that if they feel that they are women by heart, they are women* (Participant 2).

Participant 10 underscores the importance of informed decision-making and individual choice,

*Surgery is not the only way to live as a transgender person… it must be an individual choice, not influenced by peer pressure. Proper representation and documentation of reality could help many people make more informed decisions about surgery* (Participant 10).

Participant 2 highlights the need for comprehensive medical evaluation and treatment before undergoing GAS,

*Other health conditions like blood sugar levels and past medical history must be noted down and treated accordingly before proceeding to the surgery. Often it is just limited to HIV tests, this must change, All the advanced medical procedures must be made available for transwomen who opt for these surgeries* (Participant 2).

**Figure 1:**
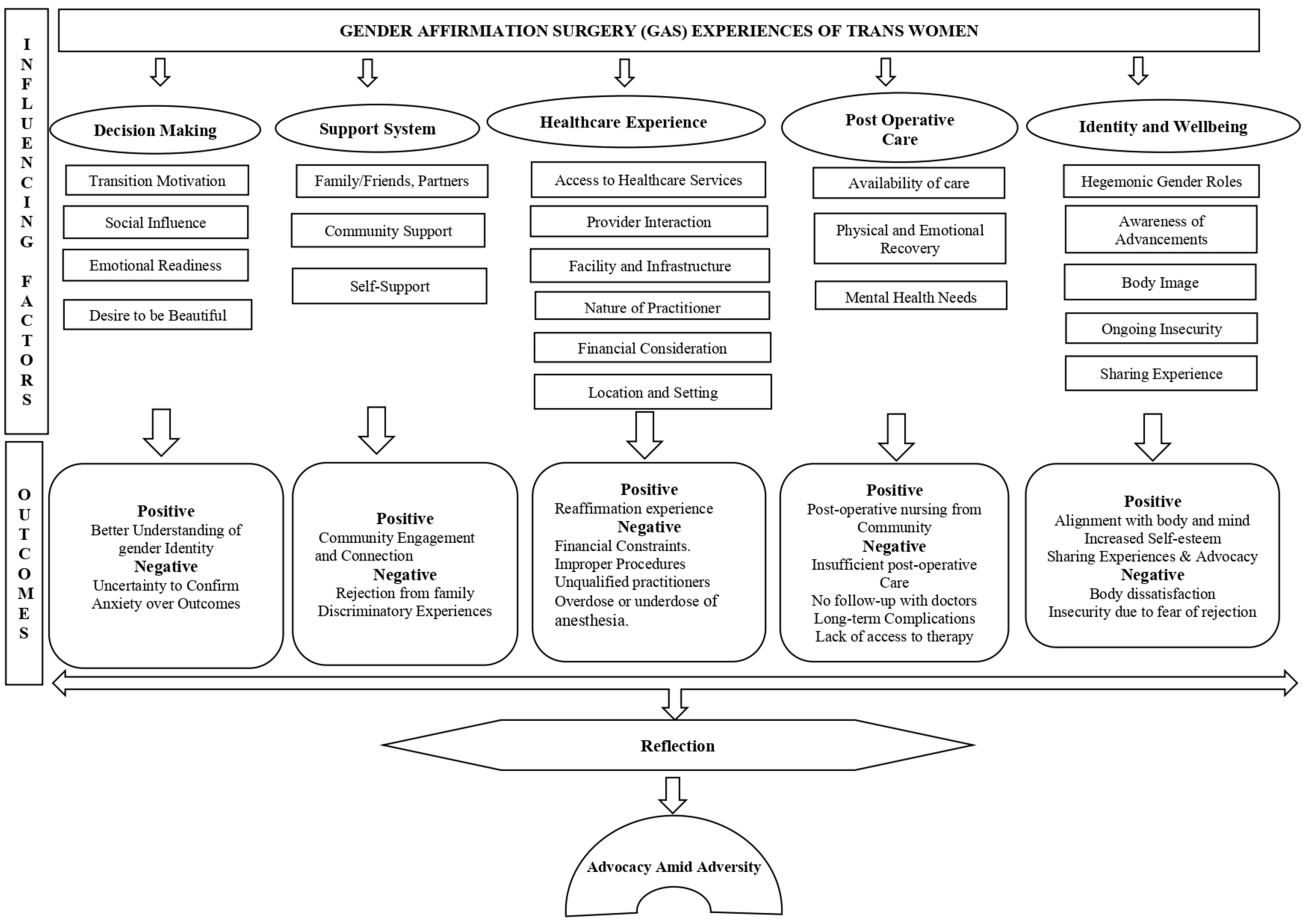
Conceptual model of trans women’s experiences with Gender Affirmation Surgery.

Serano (2007) posits that true gender equity involves dismantling both traditional and oppositional sexism, challenging the idea that transwomen must prove their identities to be recognized. Participant 2’s statement “‘if they feel that they are women by heart, they are women” reclaims transwoman’s identity and rejects the idea that surgery or external validation should define trans womanhood (Figure 1).

## Discussion

In India, the transgender community faces significant challenges, including social stigma, discrimination, and limited access to healthcare services. These challenges are compounded by socio-economic factors that further marginalize trans individuals, making it difficult for them to access necessary medical procedures, including GAS (Chakrapani, 2010). Despite the progressive legal changes, such as the Transgender Persons Protection of Rights Act (Government of India, 2019), which aims to protect the rights of transgender individuals, many still encounter barriers in accessing gender-affirming healthcare (Raj and Dubey, 2024).

Transfeminism is critical of the medicalization of trans bodies, stresses bodily autonomy, and contends that trans women are not to be compelled into medicalized transitions as a condition of gender legitimacy (Koyama, 2003). Intersectionality is embedded within transfeminism, which helps in understanding various other barriers such as class, education, and family acceptance that shape trans women’s access to healthcare.

Surgery access does not guarantee positive medical experiences, and trans women commonly find themselves facing a healthcare system that both treats and harms them through a process called iatrogenic violence (Atuk, 2024). In addition, Zengin (2016) describes “violent intimacies,” which describes how medical institutions control trans bodies employing compulsory examinations, surgical gatekeeping, and institutional contact.

### Decision making

Many trans women describe their motivation to undergo Gender Affirmation Surgery as deeply rooted in a desire to align their physical bodies with their gender identity. This motivation is often driven by years of experiencing gender dysphoria, which is caused as a result of prolonged gender incongruence marked by a persistent mismatch between an individual’s internal sense of gender and assigned sex, leading to a desire to transition and align one’s body with the experienced gender identity (World Health Organization). The perceived necessity of surgery to be seen as ‘truly’ a woman can create additional pressure.

Transfeminism criticizes this pressure, arguing that trans women should not be forced to undergo medical treatments to “validate” their womanhood and that gender should not be determined by social approval (Koyama, 2003). This critique challenges the medical gatekeeping that routinely manages access to these surgeries, contending that gender identity need not be dependent on surgical alteration. These ideals perpetuate a binary understanding of gender, with surgery viewed as a procedure to achieve authenticity of gender identity rather than a personal option.However, hegemonic femininity, which asserts that the female body must fulfill specific physical requirements, is often the driving force for trans women’s decision to have surgery (Butler, 1990).

Decision-making is also determined by intersecting factors such as financial support, improved access to healthcare, emotional preparedness, etc. (Crenshaw, 1989). Most trans women, especially from lower socio-economic classes, are coerced into unsafe, underground surgeries out of financial barriers, which perpetuates the healthcare access disparity. Transfeminism highlights how economic disparity compounds these challenges, as wealthier trans individuals can access private healthcare while poorer trans women are forced into unsafe surgical conditions or traditional castration practices (Revathi, 2010).

The lack of a consistent support network made the challenging journey even more difficult and isolating for transwomen pushing them to the ‘death world’ (Mbembe and Corcoran, 2019) forcing them to a living dead state. Furthermore, the financial burden of Gender Affirmation Surgery is significant, with many trans women struggling to afford the surgery. This factor often delays the decision-making process or compels individuals to seek alternative, sometimes risky methods of surgery.

Emotional readiness is essential for undergoing Gender Affirmation Surgery. Trans women experience fluctuating emotions, including hope, excitement, fear, and anxiety. Unfortunately, many trans women do not have access to pre-surgical counseling due to reasons such as lack of awareness, fear of judgment, and limited resources, including financial constraints, lack of qualified professionals and inadequate healthcare. While many trans women report a better understanding of their gender identity after surgery, the anxiety and uncertainty before the procedure draw attention to the importance of comprehensive pre-surgical counseling and support. Notably, although the decision to undergo surgery is often framed as an act of self-determination, medical institutions exert significant control over trans bodies.

### Social support

Support systems are an essential component of the Gender Affirmation Surgery experience for trans women, with community support networks providing emotional and practical support during and after the surgical process. However, limited family support can have serious consequences, including increased emotional distress, financial burdens, and feelings of isolation. In contrast, having a supportive partner can offer significant emotional stability and validation during a challenging transition period. Nevertheless, the persistent social stigma and discrimination against trans women can lead to negative mental health outcomes such as depression and anxiety. Transwomen also face difficulties in accessing the medical provisions provided by the state due to stigma and longer waiting hours.

Furthermore, trans women may need to rely on community support to obtain gender affirmation surgery due to financial obstacles and limited access to affordable healthcare, underscoring the necessity of targeted measures to address these systemic inequities. According to Koyama (2003) trans women are more likely to experience violence than non trans women, This is a reflection of the larger failure of cisnormative legal and medical institutions, which continue to marginalize trans bodies and place the burden of care on already vulnerable populations.

### Healthcare experience

Accessing gender-affirmation surgery can be a complex and challenging process. Many trans women who choose to undergo surgery at government hospitals often face long waiting times, stigma, and a lack of knowledgeable healthcare providers. However, even when medical care is accessible, trans women frequently encounter discriminatory treatment from healthcare providers, ranging from verbal abuse to outright denial of care (Atuk, 2024). These barriers can lead to delays in surgery, causing trans women to seek quicker but sub-standard services. The interaction between trans women and healthcare providers can significantly impact the surgical experience. Positive, affirming interactions lead to better outcomes and higher level of satisfaction. Complications of gender-affirmation surgery are both physical and psychological. These can range from surgical complications like urethral stricture and urinary infection to post-surgical depression or anxiety. Proper counseling and follow-up care are essential in alleviating these issues. Transfeminism emphasizes the importance of intersectional care, recognizing how gender identity intersects with other factors to create barriers to accessing holistic, patient-centered care that addresses both physical and emotional well-being.

These barriers in healthcare access point to the need for better access, facilities and enhanced training for healthcare providers to ensure safe and affirming care. Similar instances were recorded worldwide, as Atuk (2024) notes how doctors in Turkey refused to examine or touch trans patients, treating them as contagious rather than individuals in need of medical assistance. Zengin (2016) documented how medical professionals in Turkey perform invasive, humiliating examinations on trans women as a prerequisite for gender recognition. These practices make hospitals into sites where trans bodies are disciplined and regulated rather than healed. Similar patterns of medical scrutiny can be observed in the Indian healthcare system as well.

### Post-operative care

Post-operative care is necessary for a successful and complete recovery. However, many trans women face difficulties in accessing this care from their families. Transwomen often seek assistance from other trans women; however, they require adequate finances to afford the same. Gender affirmation surgery recovery involves both physical and emotional processes. Physically, it requires careful pain management, wound care, and management of potential complications. Emotionally, individuals find the process challenging as they adapt to their new bodies and society’s reactions to their changed appearance. Mental health support is a vital component of post-operative care as many trans women experience a range of emotions, from relief and joy to dissatisfaction and anxiety. Gender-affirming therapy and support groups can provide essential support during this time. The findings reveal that most of the trans women interviewed have not sought gender-affirming therapy post-surgery. The absence of adequate support during the post-operative period can lead to complications such as urinary tract infections, longer healing times, and in the most serious outcome, the need for re-surgery. Trans women who do not have support from their families, communities, or healthcare providers are more likely to face mental and physical challenges during their transition. Atuk’s (2024) findings illustrate how trans women in Turkey, particularly those engaged in sex work, often receive substandard or completely denied surgical care due to medical professionals’ biases. Similar experiences exist in India, where trans women report being dismissed or neglected when seeking post-surgical treatment, reinforcing medical institution’s involvement in structural violence. Integrated healthcare services that provide continuous support post-surgery are essential to address these needs.

### Identity and wellbeing

The impact of Gender affirmation surgery on identity and well-being is profound, with many trans women experiencing increased satisfaction, improved self-esteem, and alignment between body and mind (Park et al., 2022). Overall satisfaction with Gender affirmation surgery varies among trans women, depending on the alignment of expectations and outcomes. Many participants report high levels of satisfaction and relief from gender incongruence, while others struggle with the social repercussions of surgery.

Knowledge of the latest surgical techniques and outcomes plays an important role in decision-making and satisfaction. Trans women who are well-informed about advancements in surgery often feel more empowered and confident in their choices. GAS can significantly impact body image, with many trans women reporting improved self-esteem and confidence. However, some experience ongoing struggles with body image, especially if surgical outcomes do not meet their expectations or due to societal beauty standards. Despite surgery, some trans women continue to experience insecurity, often due to internalized transphobia or personal dissatisfaction with surgical outcomes. These insecurities can affect overall mental health and well-being. Sharing their experiences with others through advocacy, support groups, or public speaking helps many trans women process their journey and contribute to the broader community. This sharing also helps to reduce stigma and raise awareness about the realities of trans experiences.

Transfeminism challenges the discourse surrounding gender affirmation surgery as the purest indicator of “successful” transition, and that one’s self-value must not depend on surgical successes or social recognition (Koyama, 2003). Internalized pressure to meet cisnormative ideals of beauty can result in post-surgical discontent, noting the necessity of an expanded definition of gender affirmation that extends beyond medical procedures (Serano, 2007).

### Reflection and advocacy amid adversity

Post-surgery reflection often reveals varied emotions and self-realization. Trans women reflect on the changes in their relationships, identity, and the societal reactions they have faced. This process of self-examination and contemplation plays a key role in shaping personal growth, serving as a strong foundation for advocacy. Advocacy for trans rights, improved healthcare access, and social acceptance are integral in addressing the challenges highlighted in this study.

Many trans women who undergo GAS become advocates for better healthcare and social understanding for future generations. Their resilience and determination to improve conditions for others highlight a collective effort to challenge existing barriers and promote trans rights.

Zengin (2016) emphasizes how trans activists resist the medical and legal scrutiny imposed on their bodies, challenging the state’s violent intimacy with their gender identities. These struggles resonate with trans women’s advocacy efforts in India, where legal victories do not always yield affirming medical care.

Advocacy takes many forms, from personal mentoring to public activism, and is often driven by the adversity they have faced throughout their journey. The experiences of trans women undergoing GAS in Chennai reveal both significant progress and challenges. A more inclusive and supportive healthcare system and society could be attained by empowering their voices. The study supports the need for positive health care for trans women devoid of discrimination and stigmatization. Advocacy efforts emphasize the need for a comprehensive, inclusive approach to support trans women undergoing gender affirmation surgery (Oles et al., 2022).

The findings indicate dichotomous responses because GAS is presented as a liberating experience for gender affirmation, but the process of undergoing it through force/coercion (Participant 2) and inadequate or no aftercare (Participant 9) reveals how cisnormative systems base treatment on conformity, which questions SDG 3’s goal of “health for all.” However, the advocacy of the participant (for example, Participant 10’s call for “proper representation”) is a prime example of transfeminist resistance to reclaim autonomy in the face of institutional ignorance and harm.

## Conclusion

This study contributes to the greater discourse of research by documenting the lived experiences, agency and resilience of trans women in Chennai who have undergone various forms of gender-affirming procedures. While much of the existing scholarship focuses on the medical, psychological and legal aspects of gender transition, this study builds the gap by placing gender affirmation within a socio-cultural framework, especially through traditional castration by Thayamma within trans women community, informal surgical procedures in unauthorized clinics and hospital-based gender affirmation surgery.

The results emphasize the importance of pre-surgical counselling, support systems and access to qualified healthcare practitioners. Persistent barriers such as financial constraints, discrimination, and inadequate post-operative care reiterate the urgent need for policy reforms. This study emphasizes the resilience of trans women and the crucial role of advocacy in their journey, calling for trans-competent healthcare training for practitioners, gender-affirming care to support trans women without discrimination or stigmatization and calls for policy reforms and mental health funding. These steps help advance sustainable development goals, particularly SDG 3 (Good Health and Well-being) and SDG 5 (Gender Equality). United Nations (2015) advocating more equitable healthcare services centering on the lived experiences of transwomen. By bringing attention to non-western, community-specific gender practices, this research provides a critical narrative to dominant global perspectives on trans healthcare and identity formation. It offers insights that intersect with larger discourses on gender, identity, and community-driven advocacy, reinforcing the need for trans-inclusive policies and scholarly engagement.

### Limitations of the study

Future quantitative studies could be undertaken to address measurable outcomes such as post-surgical satisfaction, which is the limitation of the current study. This study exclusively focuses on trans women and hence does not capture the experiences of trans men or non-binary individuals, which could be a scope for future research.

## Data Availability

Due to the sensitive nature of the study and participants privacy, the raw data of this article cannot be made publicly available. Requests to access the datasets should be directed to the corresponding author.
